# Characterizing 3T3-L1 MBX Adipocyte Cell Differentiation Maintained with Fatty Acids as an In Vitro Model to Study the Effects of Obesity

**DOI:** 10.3390/life13081712

**Published:** 2023-08-09

**Authors:** Noshin Mubtasim, Lauren Gollahon

**Affiliations:** Department of Biological Sciences, Texas Tech University, 2500 Broadway, Lubbock, TX 79409, USA; nmubtasim@gmail.com

**Keywords:** adipocyte, obesity, 3T3-L1 MBX, adipocytokine, adipogenesis, in vitro fat cell model

## Abstract

The increasing prevalence of obesity has prompted intensive research into understanding its role in pathogenesis and designing appropriate treatments. To determine the signals generated from the interaction of fat cells with a target organ, a reliable white adipocyte model in vitro is needed. Differentiated fibroblasts are the most extensively studied using in vitro cell models of white adipocytes. However, it can be argued that differentiated fibroblasts minimally recapitulate the consequences of obesity. Here, we describe 3T3-L1 MBX cells as a culture model for studying obese adipocytes and their effects. Differentiation of 3T3-L1 MBX cells was at first optimized and then maintained in the presence of fatty acids cocktail combination to induce the obese condition. Lipid accumulation and adipokine secretion profiles were analyzed. Results showed that fatty acid-maintained, differentiated 3T3-L1 MBX cells had significantly greater accumulation of lipids and significant changes in the adipokine secretions in comparison to differentiated 3T3-L1 MBX cells maintained in medium without fatty acids. To elucidate the molecular changes associated with adipogenesis and lipid accumulation profile of 3T3-L1 MBX cells, we have also explored the expression of some of the regulatory proteins related to the development and maintenance of adipocytes from the preadipocyte lineage.

## 1. Introduction

In the last 40 years, one-third of the world’s countries have been identified with significant percentages of obese populations [1]. Obesity is a chronic, low-grade metabolic disorder defined by excessive fat accumulation in white adipocytes due to increased calorie intake and reduced calorie expenditure [1]. Physiologically, white adipose tissue (WAT) is the body’s energy storage organ, where unused body fat gets stored as triglycerides [2]. Also, WAT releases proteins known as adipokines that have paracrine and endocrine effects [3,4]. However, due to the excessive fat accumulation in obesity, there is an infiltration of proinflammatory immune cells in WAT, followed by changes in WAT adipokine secretion profiles [5]. In addition to that, due to other complex pathophysiological consequences of obesity (i.e., changes in the gut microbiome, insulin resistance, oxidative stress, and inflammation), a susceptibility to develop different comorbid, diseased conditions such as Type-2 diabetes mellitus, cancer (liver, colon, breast, ovary, prostate), fatty liver, hypertension, Alzheimer disease, depression, and asthma, increases [1,6,7,8]. The increasing prevalence of obesity has prompted intensive molecular biology research worldwide to better understand the underlying mechanisms of obesity toward the pathogenesis of non-communicable diseases.

To tackle the outcomes of obesity and its associated disorders more effectively, elucidating the effects at the cellular and molecular level is critical. Generally, these first steps involve in vitro research. Differentiated fibroblast cells are widely used as a fat cell model for in vitro investigation into the signaling effects generated from the interaction of fat cells with the target organ cell model.

### The Differentiation Process of 3T3-L1 Cells

3T3-L1 cells comprise the most extensively studied in vitro cell model of white adipocytes [8,9,10,11,12,13,14,15,16,17]. 3T3-L1 is clonally derived from 9-day-old Swiss 3T3 mouse embryos immortalized in 1960 [9]. They are fibroblasts that can be induced to differentiate into cells that have a morphological and biochemical resemblance to white adipocytes [18]. Prior to differentiation, they are referred to as preadipocytes. The pharmacologic stimulation (Table 1) for the differentiation of 3T3-L1 cells in culture contains the cAMP elevating agent methyl isobutyl xanthine (IBMX), a glucocorticoid dexamethasone, and insulin (10). Exposure of the preadipocytes to the pharmacological cocktails triggers mitotic clonal expansion, which enables growth-arrested fibroblast cells to enter mitosis (limited to 1–2 rounds), while synergistically activating a transcriptional factors’ cascade relating to adipogenesis [19]. The activation of cAMP signaling pathways by IBMX activates the CREB protein through phosphorylation [19]. Furthermore, this phosphorylated CREB protein, together with the c/EBPβ gene promoter, induces the endogenous expression of C/EBPβ [19]; 18–24 h post differentiation induction, synchronous entry of 3T3-L1 cells into S phase occurs after the sequential phosphorylation of c/EBPβ transcriptionally activates the expression of C/EBPα and PPARγ genes by binding to their regulatory elements [19]. C/EBPα and PPARγ then play a role as transcription factors, and together they transactivate large groups of adipocyte-specific genes for the terminal differentiation of preadipocyte cells to adipocyte phenotype [19,20,21]. Early differentiation signaling by insulin is mediated via the IGF-1 receptor-mediated activation of PI3K and MAPK signaling pathways [22]. Following induction, the preadipocytes lose their fibroblast morphology and start acquiring the characteristic adipocyte-like spherical morphology and accumulating lipids [19]. Differentiated 3T3-L1 cells have been widely used as the in vitro fat cell model to decipher the molecular mechanisms behind obesity-associated chronic disease pathogenesis, progression, and treatment [10,11,12,13,14,15,16,17,23].

Exposure of the 3T3-L1 fibroblasts to the pharmacological cocktail of differentiation components for 3 days also endows them with the capability of accumulating lipids as triglycerides. The terminal phase of adipocyte differentiation is marked by the rise in the enzymes involved in de novo lipogenesis [18]. This is partly driven by an increase in adipocyte sensitivity to insulin after differentiation. This occurs through the intracellular trans-localization of GLUT4 receptors from the cytosol to the plasma membrane and GLUT4-mediated glucose uptake [25]. Insulin stimulation also transcriptionally regulates the expression of the enzyme fatty acid synthase (FAS), which is involved in the synthesis of fatty acids through de novo lipogenesis [25]. Adipogenic differentiation with the pharmacological cocktail also synchronously increases the expression of lipoprotein lipase after differentiation. This enzyme is involved in the breakdown of exogenous lipoproteins into fatty acids [27]. The uptake of fatty acids by adipocytes is further fostered by insulin-stimulated, intracellular localization of fatty acid transporter proteins such as CD36 and the fatty acid transport protein-1 (FATP1) to the plasma membrane [25]. Fatty acids from these two sources are then esterified and deposited as triglycerides in the lipid droplets of differentiated 3T3-L1 cells [28].

Obesity is correlated with an increased concentration of free fatty acids in blood circulation [29,30]. Digestively, consumed fat breaks down into fatty acids, and any excess accumulates in white adipocytes as triglycerides after meeting physiological needs [31]. When white adipocytes become oversaturated with triglycerides, the breakdown of triglycerides into fatty acids increases circulating levels. Additionally, excess fatty acids also accumulate in vital organs such as the liver, pancreatic beta cells, skeletal muscles, and heart as ectopic fat [31,32]. This underscores the profound effect of excess fatty acids on critical organs. However, it is difficult to design experiments based on these in vivo observations for in vitro, mechanistic research. Thus, the current investigation has addressed this obstacle by analyzing the molecular events of obesity-associated changes on the 3T3-L1(CL-173) clonal derivative 3T3-L1 MBX cells, with the combinatorial treatment of excess fatty acids, inducing an obese condition for its suitability as in vitro study model for obesity-associated pathogenesis study. While some studies report the adipogenic changes in 3T3-L1 cells with different classes of fatty acids individually [33,34,35,36,37], differentiation to mature and obese adipocytes in 3T3-L1 MBX cells remains to be explored. Moreover, there is no reported data to date that has addressed the effect of a fatty acids cocktail on the secretion of adipokines and chemokines in 3T3-L1 MBX cells. The results from this study show that 3T3-L1 MBX can serve as an in vitro, obese fat cell model for obesity research studies. The study was accomplished by first optimizing the differentiation of 3T3-L1 MBX cells into mature and then obese adipocytes and then analyzing the lipid accumulation and adipokine secretions profile of differentiated 3T3-L1 MBX cells, maintained in the presence or absence of a fatty acids cocktail. The present study has also explored the expression of some of the regulatory proteins controlling the development to maintenance of differentiated 3T3-L1 MBX adipocytes from the preadipocyte fibroblast lineage.

## 2. Materials and Methods

### 2.1. Cell Cultures

3T3-L1 MBX (ATCC^®^ CRL-3242), mouse embryonic fibroblast, was purchased from American Type Culture Collection. Cells were grown in Dulbecco’s Modified Eagle’s Medium (DMEM) supplemented with high glucose and glutamax (Thermo Fisher Scientific, Waltham, MA, USA; Catalog # 10569010) in the presence of 10% fetal bovine serum (Atlas Biologicals, Fort Collins, CO, USA; Lot# E10J20A1) and 1% streptomycin plus penicillin (P/S) (Thermo Fisher Scientific; Catalog # 15140122). After cells reached ~80% confluency, the pharmacological stimulators for adipogenesis (Methyl-3-Isobutyle xanthine (Sigma-Aldrich, St. Louis, MO, USA; Catalog # I7018), insulin (Sigma-Aldrich; Catalog # I0516), dexamethasone (Enzo Life Sciences, Farmingdale, NY, USA; Catalog# BML-EI126-0001), and rosiglitazone (Cayman Chemical, Ann Arbor, MI, USA; Catalogue# 71740), were added to induce differentiation. In the differentiation cocktails, rosiglitazone was added as the 4th pro-differentiating agent. For the purposes of this study, the concentrations of insulin and dexamethasone were increased and optimized based on evaluating the lipid accumulation profile of differentiated 3T3-L1 MBX using oil-red O stain (Sigma-Aldrich; Catalog# O1391). Furthermore, the number of days of differentiation induction was also optimized based on evaluating the lipid accumulation profile of differentiated 3T3-L1 MBX by oil-red O stain.

A 5 mM stock solution of dexamethasone was prepared by dissolving 5 mg of dexamethasone powder in 12.75 mL ethanol. This stock solution was aliquoted and preserved at −80 C. From this stock solution, an intermediate solution of dexamethasone at a 1 mM concentration was prepared by mixing 2 mL of dexamethasone from the 5 mM stock in 8 mL of complete DMEM media. This intermediate stock was preserved at −20 C, and from this solution, the required volume was added to the final differentiation cocktail solution.

Insulin was purchased as a stock conc. of 10 mg/mL. This was diluted to 10 µg/mL to differentiate 3T3-L1 MBX cells.

### 2.2. Optimizing for Differentiation

To optimize the concentration of differentiation components for 3T3-L1 MBX differentiation, four experimental variable conditions were performed.

1. Concentration of compounds used for 3T3-L1differentiation: Methyl Isobutyl xanthine (0.5 mM); Dexamethasone (0.25 µM); Insulin (1 µg/mL); Rosiglitazone (2 µM) [38].
2. Increasing only the concentration of Insulin: Insulin (10 µg/mL); Methyl Isobutyl Xanthine (0.5 mM); Dexamethasone (0.25 µM); Rosiglitazone (2 µM)
3. Increasing only the concentration of Dexamethasone: Dexamethasone (1 µM); Methyl Isobutyl xanthine (0.5 mM); Insulin (1 µg/mL); Rosiglitazone (2 µM)
4. Increasing the concentrations of both Insulin and Dexamethasone: Insulin (10 µg/mL); Dexamethasone (1 µM); Methyl Isobutyl xanthine (0.5 mM); Rosiglitazone (2 µM)

In addition, the time for inducing differentiation (2 vs. 3 days) in 3T3-L1 MBX cells with the improved differentiation cocktail was also optimized based on their lipid accumulation profiles stained with oil-red O staining.

### 2.3. Optimized Differentiation Condition

3T3-L1 MBX was grown to 80% confluence in DMEM containing 10% FBS and 1% P/S. Following this, the cells were induced to differentiate for 72 h (~3 days) with DMEM containing 10% FBS, 1% P/S, 0.5 mM Methyl-3-Isobutyle xanthine, 10 µg/mL insulin, 1 µM dexamethasone, and 2 µM rosiglitazone. For the next 3 days, the differentiated cells were maintained in DMEM containing 10% FBS, 1% P/S, and 1 µM insulin. On Day 6, the medium was changed to DMEM containing 10% FBS and 1% P/S and refreshed on every alternate day.

### 2.4. Oil-Red O Staining

3T3-L1 MBX adipocytes were washed twice with phosphate-buffer saline (PBS) and then fixed for 20 min in 4% paraformaldehyde. After discarding the paraformaldehyde, the fixed cells were again washed with PBS twice and subsequently covered with the working solution of oil-red O stain for 15 min at room temperature. The working solution of oil-red O was prepared by adding 3 parts of oil-red O stock with 2 parts of distilled water, keeping it undisturbed for 10 min, and then filtering it with coarse filter paper. The working solution is stable for 2 h and should be prepared 15 min before use. After staining, the excess dye was washed with distilled water until the background was clear. To image the oil accumulation, an EVOS XL Core imaging system (Thermo Fisher Scientific; Catalog# AMEX1000) was used. For quantitative analysis, the stained dye was extracted with 100% isopropanol for 15 min at room temperature with gentle rocking and quantified using a Bio-Tek Synergy H1 Microplate Reader H1M at 518 nm. For quantitative analysis, 100% isopropanol was used as the background control.

### 2.5. Fatty Acids Treatment

The following fatty acids were chosen for the study to evaluate their effect on 3T3-L1 MBX cell’s lipid accumulation and secretion profile [37]: monounsaturated (oleic acid), polyunsaturated (linoleic acid) and saturated (palmitic acid). To determine the appropriate non-toxic concentration of fatty acids cell viability assay analysis, the optimal cell density of differentiated 3T3-L1 MBX was optimized for a 96-well plate using the MTT assay kit (CyQuant^TM^ MTT Cell Viability Assay, Thermo Fisher Scientific, Waltham, MA, USA; Catalog# V13154); 4000 to 4500 cells were seeded on 96-well plates and incubated for 24 h for attachment. After attachment, the cells were grown in the differentiation cocktail for adipogenesis for 3 days. After induction of differentiation, cells were incubated with maintenance media containing DMEM with insulin. At this point, in addition to insulin, cells were treated with a range of concentrations of oleic acid, linoleic acid, and palmitic acid separately in their respective well plate for 24 h. The concentration range of each fatty acid was 0.1 mM, 0.25 mM, 0.35 mM, 0.5 mM, 0.75 mM, 0.85 mM, 1 mM). The wells containing no cells serve as a blank, and differentiated 3T3-L1 MBX cells, maintained in DMEM with no fatty acids, served as the negative control. The viability of 3T3-L1 MBX cells in each condition was determined using an MTT assay.

Bovine Serum Albumin (BSA) conjugated oleic (Catalog# O3008; Lot #SCLF1158) and linoleic (Catalog# L9530; Lot #SLCC5450) fatty acid in liquid form were purchased from Sigma Aldrich. Palmitic acid (Catalog# P5585; Lot #SLCF9094) was purchased as a powder formulation from Sigma Aldrich; 500 mM concentration of stock palmitic acid solution was prepared by dissolving 128.1 mg of palmitic acid powder in 100% ethanol at first and then heating it at 70 °C; 100 µL of heated palmitic acid solution was dissolved in 10 mL of 10% BSA solution prepared in 150 mM of sodium chloride (NaCl) solution. The solution was incubated at 55 °C for an hour, and later, it was stored at −20 °C. The final working concentrations were non-toxic and greater than their reported physiological concentration (oleic acid, 1–10 µM; linoleic acid, 1–10 µM; palmitic acid, 1–100 µM) [37].

### 2.6. MTT Assay Analysis

12 mM stock of MTT was prepared by adding 1 mL of sterile PBS to one 5 mg vial of MTT on the day of analysis and vortexed until dissolved. The solution was filtered to remove large particles. Media was carefully cleared from each well, and 100 µL of fresh media was added; 10 µL of 12 mM MTT stock solution was added to each well and incubated at 37 °C overnight. The next day, the SDS-HCl solution was prepared by adding 10 mL of 0.01 M HCl to the manufacturer-supplied bottle of SDS. It was also vortexed or sonicated until dissolved and used promptly. Later, 100 µL of the SDS-HCl solution was added to each well containing MTT and pipetted up and down to dissolve the purple crystals. The 96-well plates were incubated at 37 °C for 4 to 5 h in a humidified chamber. Readings of the absorbance at 570 nm were measured using a Bio-Tek Synergy H1 Microplate Reader H1M.

### 2.7. Adipocytokine Testing

On day 10 post differentiation, the used medium was discarded, and DMEM medium supplemented with 1% antibiotics and 0.5% FBS was added to the differentiated cells. The differentiated adipocytes were cultured for an additional 24 h to allow for adipokine secretion and media conditioning. The next day, the media was collected, centrifuged, filtered, and stored at −80 °C for later use. The adipocyte secretion profiling of the mature and obese 3T3-L1 MBX-derived media, 11 days after the initiation of differentiation, was perfomed using a proteome profiler mouse adipokine array kit (R & D System, Catalog# ARY013), following the manufacturer’s protocol. Chemiluminescence was detected with the Odyssey^®^ Fc Imaging System by LI-COR Biosciences with 8 min exposure time.

### 2.8. Protein Extraction and Quantification

Lysis buffer for protein extraction was prepared by mixing Pierce RIPA lysis buffer (Lot# XG348655, Thermo Scientific) with Halt Protease and Phosphatase inhibitor (100X), and 0.5 M EDTA (catalog# 78420, 78420, 87886; Thermo Fisher Scientific) Cocktail. The inhibitor cocktail contains a broad spectrum of protease inhibitors (AEBSF, aprotinin, E-64, bestatin, leupeptin, and pepstatin A) and phosphatase inhibitors (sodium fluoride, sodium orthovanadate, sodium pyrophosphate, and beta-glycerophosphate). For every 500 µL of lysis buffer, 5µL of 100X protease, phosphatase, and EDTA was added to a final concentration of 1X.

To prepare the whole-cell lysate of 3T3-L1 MBX cell from Day 0, Day 3, Day 6, and Day 11 (adipocytes w/o fatty acid treatment), at first, the existing media on the culture dish was discarded, and the cells were washed in cold FBS-free DMEM media. Followed by the cold FBS-free DMEM media was discarded, and 500 µL of ice-cold lysis buffer was added. The culture dish was put at −80 °C for 3 min. Afterward, the cells were scraped using ice cold plastic cell scrapper and collected in a microcentrifuge tube. The contents in the centrifuge tube were agitated for 15 min and then were centrifuged at 12,000 rpm for 15 min at 4 °C. After centrifugation, the cell pellet was discarded, and the cell supernatant was collected as a whole cell lysate. The BCA protein assay was used to determine the protein concentration of the whole cell lysate of the different samples of 3T3-L1 MBX from different days of differentiation. Reference standard protein (albumin) concentrations were made as per the manufacturer’s instruction described on the BCA assay protocol included in the Pierce BCA assay kit (Thermo Scientific); 200 µL of protein analysis solution was added to 25 µL of standard and sample proteins, each in duplicates, as per the instructions mentioned in the protocol. Finally, reference standard and sample protein concentrations were quantified using a 562 nm laser on Bio-Tek Synergy H1 Microplate Reader H1M.

### 2.9. SDS-Polyacrylamide Gel Electrophoresis and Immunoblotting

The amount of lysate required to load 40 µg of protein per lane from 3T3-L1 MBX protein sample from different days of differentiation was calculated. The volume of lysate was then mixed with a 6X Laemmli sample in a 5:1 ratio. The rest of the volume of the lysate was adjusted using RIPA lysis Buffer to make the loading sample. The prepared loading sample was then denatured by heating them at 95 °C for 5 min, and 40 µg of denatured lysate was loaded to each well of hand-cast SDS-polyacrylamide gel. Hand-cast polyacrylamide gel, used to run the denatured lysates from 3T3-L1 MBX protein sample from different days of differentiation, was made of 8–10% gradient resolving gel and 4% stacking gel. For high molecular weight protein (MW > 250 kDa), 6–10% gradient resolving gel and 4% stacking gel were used. Ezrun Prestained Rec Protein Ladder was (Lot# 0015688; Fisher Bioreagents) used as a reference ladder. The denatured lysates were resolved on the gradient polyacrylamide gel at 120 V until 17 kDa protein reached the bottom of the gel. A wet transfer technique was undertaken to transfer the protein from the gradient polyacrylamide gel to the nitrocellulose membrane (Lot # XE3443891; Thermo Scientific). The wet transfer was performed in an ice bath with 1X transfer buffer (25 mM Tris; 192 mM glycine) at 110 V for 60 min. For high molecular weight protein (MW > 250 kDa), the denatured samples were run for 65 min at 110 V. After transfer, Ponceau S solution was used to assess the validity of protein transfer. Afterward, nitrocellulose membranes were blocked with 3% bovine serum albumin blocking buffer made in 1X TBS-Tween (Trizma HCl, NaCl, ultra-pure water, Tween 20) buffer for an hour. Following blocking, nitrocellulose membranes were incubated with primary antibodies at 1:1000 dilution overnight. To see the expression of proteins in 3T3-L1 MBX cells from different days of differentiation, AMPKα Rabbit mAb (Product # 5831), Phospho-AMPK-α Rabbit mAb (Product # 2535), CBP Rabbit mAb (Product # 7389), GCN5L2 Rabbit mAb (Product # 3305), PPAR-γ Rabbit mAb (Product# 2435) SirT1 Rabbit mAb (Product # 2496), RXRα Rabbit mAb (Product# 3085) primary antibodies from Cell Signaling Technology were used at 1:1000 dilution for overnight incubation. After the primary antibody incubation, the nitrocellulose membranes were washed with 1X TBS-Tween buffer 3 times for 5 min each. Later, the membranes were incubated with Goat Anti-Mouse IgG HRP conjugated (Lot# A1014; Santa Cruz Biotechnology), and Goat Anti Rabbit IgG HRP conjugated (Product # 7074, Cell Signaling Technology) secondary antibody for 90 min. Membranes were further washed prior to ECL exposure and developed using a Licor Imaging system. The relative protein densities of the same protein from different samples of 3T3-L1 MBX cells were analyzed using Image J software. The developed membrane was restored using Restore PLUS Western Blot Stripping Buffer (Lot# UJ291024, Thermo Scientific); if any other protein expression is desired to watch on the same membrane. The restored membrane was blocked, washed in between the primary and secondary antibody incubation, and developed for another protein repeating the experimental steps in the same way mentioned above.

### 2.10. Statistical Analysis

The statistical interpretation of the data was performed using GraphPad Prism 9 software (GraphPad Software Inc., San Diego, CA, USA). Each experiment was conducted independently 3 times with replicates, and the resultant data was expressed as mean ± standard error. The statistical significance between the treatment group and control was determined using One-way Analysis of Variance (ANOVA) and paired sample *t*-test (Adipokine array test). Tukey’s post hoc test was employed for multiple comparisons of group means in between. Dunnett’s multiple comparison compares means from several experimental groups against the means of a control group. It was employed to observe the differences in (a) the dose range effect of different fatty acids against the negative control and (b) regulatory protein expression in differentiated 3T3-L1 MBX cells against non-differentiated 3T3-L1 MBX fibroblast cells. Overall, *p*-value <0.05 was considered statistically significant.

## 3. Results

### 3.1. Increased Concentrations of Dexamethasone and Insulin Increased the Amount of Accumulated Lipids in 3T3-L1 MBX

**Figure 1:**
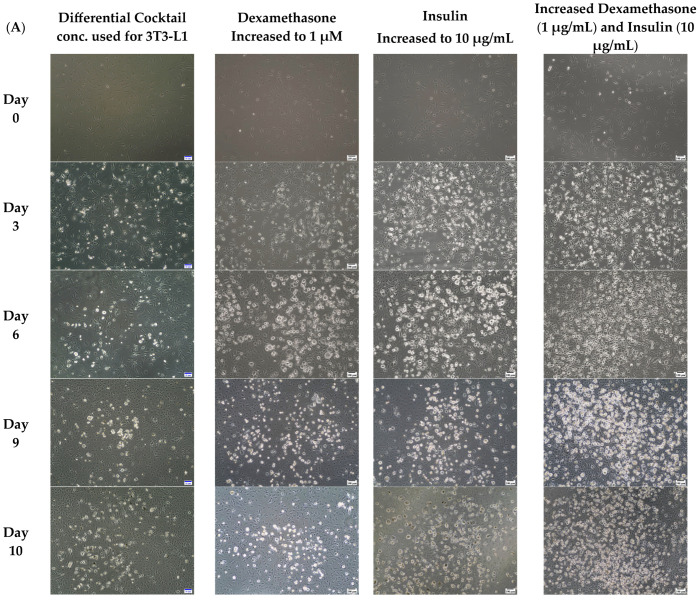

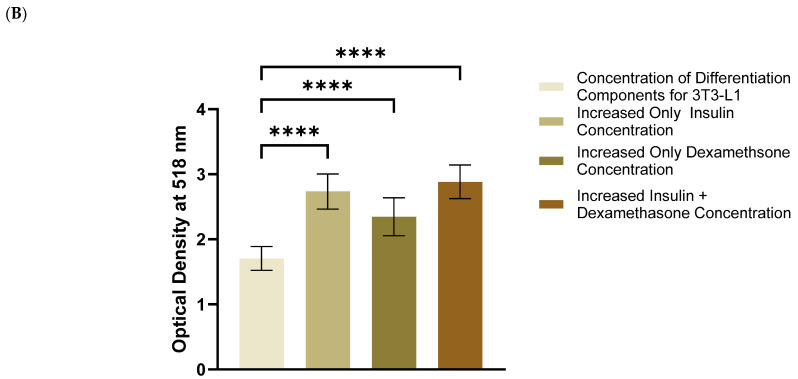
Quantitation of accumulated lipids with oil-red O stain in differentiated 3T3-L1 MBX cells on Day 11 with increased concentrations of insulin and dexamethasone. (**A**) Cell culture images demonstrating the efficiency of differentiation in 3T3-L1 MBX cells with increased concentrations of dexamethasone (1 µM) and insulin (10 µg/mL) over 10 days post-initiation of differentiation. (**B**) Quantitative evaluation of the efficiency of differentiation with the dexamethasone (1 µM) and insulin (10 µg/mL) in 3T3-L1 MBX on Day 11 post-initiation of differentiation, using oil-red O stain. Each data point represents the average measure of optical density for oil-red O stain using a plate reader from 3 replicates of 3 independent experiments. The statistical significance between the treatment group and control was determined using One-way Analysis of Variance (ANOVA), and Tukey’s post hoc test was employed for multiple comparisons between group means. A *p*-value < 0.05 was considered statistically significant. Four asterisk (****) indicates a *p*-value less than 0.0001.

The initial goal was to optimize the differentiation of 3T3-L1 MBX cells by changing the concentration of insulin and dexamethasone. Approximately 250K, 3T3-L1 MBX cells were seeded in 6-well plates and grown to ~80% confluence before inducing differentiation for each condition. 3T3-L1 cells (ATCC) are differentiated with methyl isobutyl xanthine (0.5 mM); dexamethasone (0.25 µM); insulin (1 µg/mL); rosiglitazone (2 µM) [38]. These same concentrations were not found to be effective in differentiating 3T3-L1 MBX cells based on the quantitative measure of accumulated lipids determined with oil-red O. Thus, the concentration of two of the differentiation components, insulin and dexamethasone, was evaluated to optimize the differentiation of 3T3-L1 MBX. The efficiency of differentiation was evaluated on Day 11 after the initiation of differentiation, using oil-red O stain. The differentiation was also monitored and captured through microscopic imaging for 10 days post-initiation of differentiation. When the concentration of dexamethasone only was increased from 0.25 µM to 1 µM, the oil-red O stained 3T3-L1 MBX cell fat accumulation was significantly increased quantitatively compared to the control. Figure 1 shows that when only the concentration of insulin was changed from 1 µg/mL to 10 µg/mL, similar significant changes in lipid accumulation were also observed in 3T3-L1 MBX cells. However, when both the concentrations of dexamethasone and insulin were increased from 0.25 µM to 1 µM and 1 µg/mL to 10 µg/mL, respectively, the oil-red O stain positive cells increased significantly from the control and the two components individually. Furthermore, imaging of differentiating 3T3-L1 MBX cells over the 10 days post-initiation of differentiation revealed close to 90% differentiation in the cultured cell population. Thus, by increasing the concentration of both insulin and dexamethasone, the 3T3-L1 MBX cells were found to have maximum differentiation. This new, optimized concentration of dexamethasone (1 µg/mL) and insulin (10 µg/mL) is recommended as the new concentration of those two differentiation components for 3T3-L1 MBX cells and was used at these concentrations in all the subsequent validation experiments of this current study.

### 3.2. Three-Day Cycles of Differentiation Induction Increased the Amount of Accumulated Lipids in 3T3-L1 MBX Significantly More than Two-Day Cycles

The second experimental goal was to evaluate the time for inducing differentiation in 3T3-L1 MBX cells (2 days vs. 3 days) with the optimized differentiation cocktail determined in experiment 1. Approximately 250K 3T3-L1 MBX cells were seeded in 6-well plates for each condition. Cells were grown to ~80% confluence and were differentiated using methyl isobutyl xanthine (0.5 mM); dexamethasone (1 µM); insulin (10 µg/mL); rosiglitazone (2 µM) for 2 days or 3 days. There was no technical control to this experiment since the aim of the experiment was to explore the changes in 3T3-L1 MBX cells if they were kept in the newly optimized concentrations of the differentiation cocktail for 2 vs. 3 days. The efficiency of differentiation induction was evaluated based on the amount of accumulated lipids in 3T3-L1 MBX on Day 11 post-initiation of differentiation using oil-red O stain. This transition was also monitored and captured with microscopy for 9 days post-initiation of differentiation.

**Figure 2:**
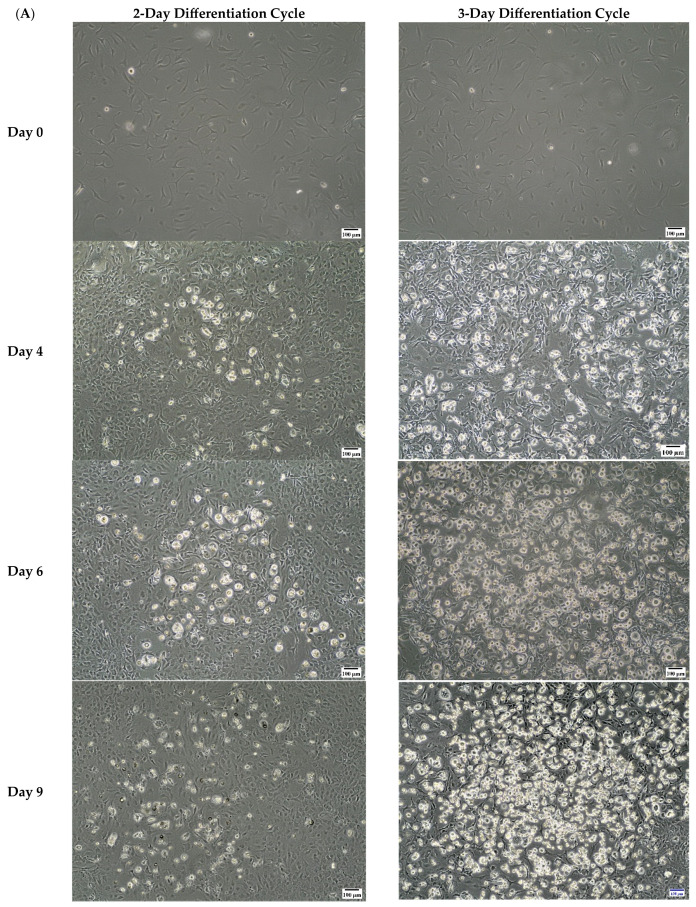

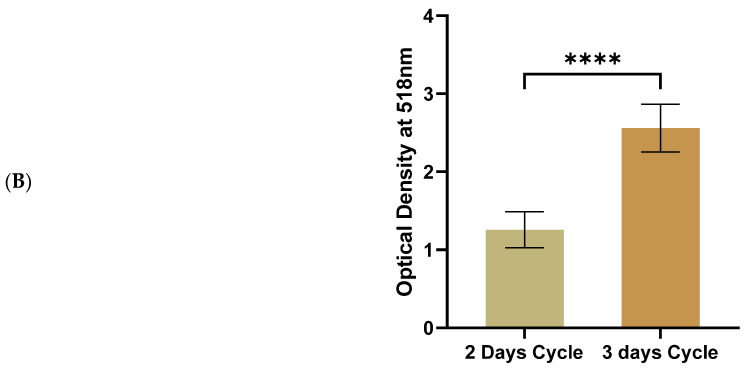
Evaluation of 3-day cycles for differentiation induction. Three-day cycles increased the amount of accumulated lipids in 3T3-L1 MBX significantly over 2-day cycles. (**A**) Photomicrographs of the difference in 3 vs. 2-day cycles for inducing differentiation in 3T3-L1 MBX over 9 days. (**B**) Quantitative evaluation of the efficiency of 3 vs. 2-day cycles for inducing differentiation in 3T3-L1 MBX cells measured on Day 11 post-initiation, using oil-red O stain. Each data point represents the average measure of optical density of oil-red O stain using a plate reader in 3 replicates for 3 independent experiments. The statistical significance between the treatment group and the control was determined using an unpaired *t*-test. A *p*-value < 0.05 was considered statistically significant. Four asterisk (****) indicates a *p*-value less than 0.0001.

In Figure 2, the results showed that 3-day cycles produced significantly higher amounts of accumulated lipids in comparison to 2-day cycles, conventionally used with 3T3-L1 induction. In addition, the 3T3-L1 MBX cells were visually observed to exhibit ~90% differentiation with 3-day cycles compared to less than ~50% for 2-day cycles. Results suggest that by inducing differentiation in 3T3-L1 MBX cells with the newly optimized differentiation cocktail for 3 days, the 3T3-L1 MBX cells differentiation increased to nearly 100%. Thus, 3-day cycles were used to induce differentiation in 3T3-L1 MBX cells in subsequent experiments. The results are shown in Figure 2.

### 3.3. Determining the Optimum Dose of Fatty Acids for Differentiating 3T3-L1 MBX Cells to the Obese Condition

**Figure 3:**
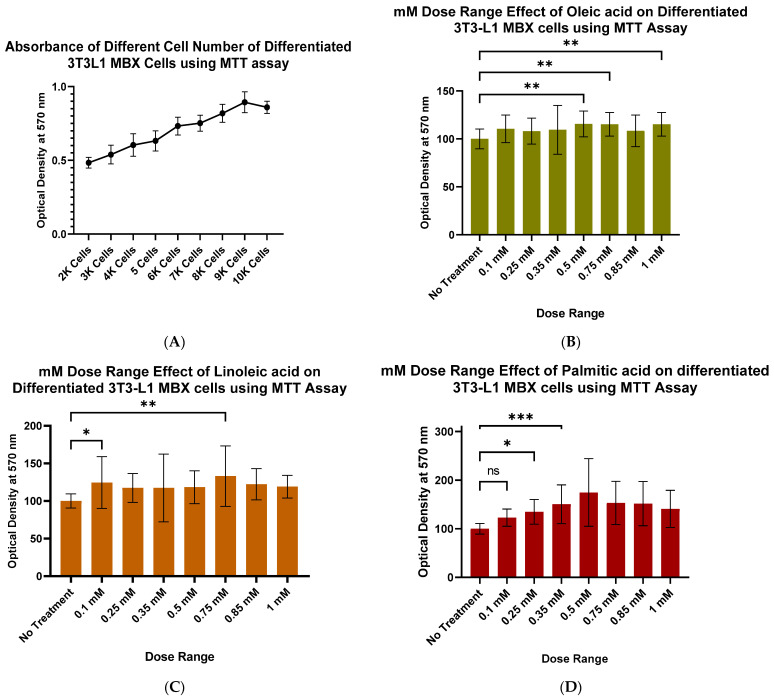
Determination of the optimum non-toxic dose of individual fatty acids on differentiated 3T3-L1 MBX cells. (**A**) Cell viability assay determining the appropriate seeding density of 3T3-L1 MBX cell in 96-well plates for the consecutive cell viability assay against individual fatty acids for their variable mM dose range on differentiated 3T3-L1 MBX cells. mM dose range effect of (**B**) oleic acid, (**C**) linoleic acid and (**D**) palmitic acid on differentiated 3T3-L1 MBX cells for 24 h. Each data point represents the average measure of optical density of formazan crystal formed during MTT assay using a plate reader in 6 replicates for 3 independent experiments. The statistical significance between the treatment group and control was determined using One-way Analysis of Variance (ANOVA), and Dunnett’s multiple comparison test was employed for comparing all the dose range effects against negative control. A *p*-value < 0.05 was considered statistically significant. One asterisk (*) indicates *p* ≤ 0.05, two asterisk (**) indicates *p* ≤ 0.01, and three asterisk (***) indicates *p* ≤ 0.001. *p*-value > 0.05 was considered statistically nonsignificant (ns).

To select the optimum non-toxic concentration of excess fatty acids for driving differentiated 3T3-L1 MBX cells to the obese condition, cell viability of the differentiated 3T3-L1 MBX cells was evaluated over a range of fatty acids and doses using the MTT assay. The results are shown in Figure 3. As a baseline, cell viability was determined on a wide range of 3T3-L1 MBX cells (2 k–10 k) on 96-well plates to find the appropriate seeding density for differentiation. For this study, 4500 cells were selected due to the differentiation properties of preadipocytes. Cell-to-cell contact-induced growth inhibition or confluency is not a prerequisite for adipocyte differentiation. Also, widely scattered cells fare better with the accumulating lipids as they convert into spherical adipocyte cells in later stages of differentiation.

3T3-L1 MBX cells were seeded at a density of 4500 cells per well in 96-well plates. On the 2nd day, differentiation was induced with Methyl Isobutyl xanthine (0.5 mM); Dexamethasone (1 µM); Insulin (10 µg/mL); Rosiglitazone (2 µM) for 3 days. Following this differentiated 3T3-L1 MBX cells were treated with a wide range of fatty acids alone or as a cocktail of oleic, linoleic, and palmitic acid. After 24 h of fatty acid treatment, the different doses of oleic acid, linoleic acid, and palmitic acid did not have any effect on the cell viability of differentiated 3T3-L1 MBX cells (Figure 3). Among the dose ranges, 0.75 mM oleic acid, 0.85 mM linoleic acid, and 0.1 mM palmitic acid were selected for further study due to the smaller deviation in their standard error bars.

### 3.4. Differentiation in the Presence of the Fatty Acids Combination, Increased the Lipid Accumulation Profile of 3T3-L1 MBX, Driving the Obese Condition

**Figure 4:**
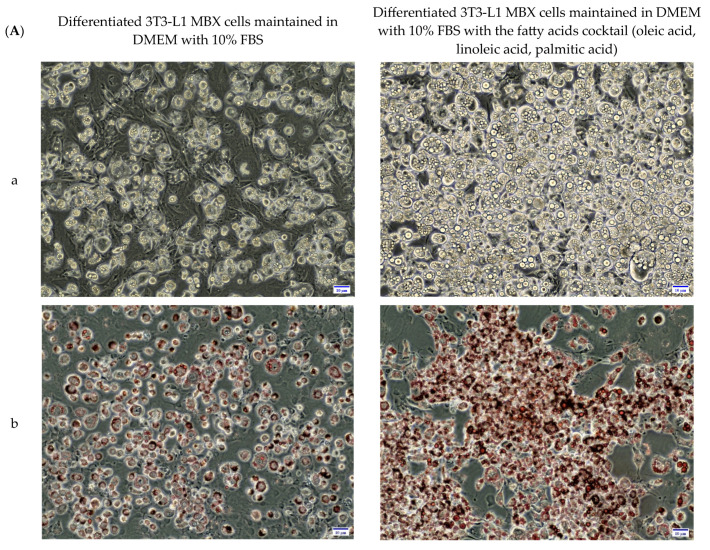

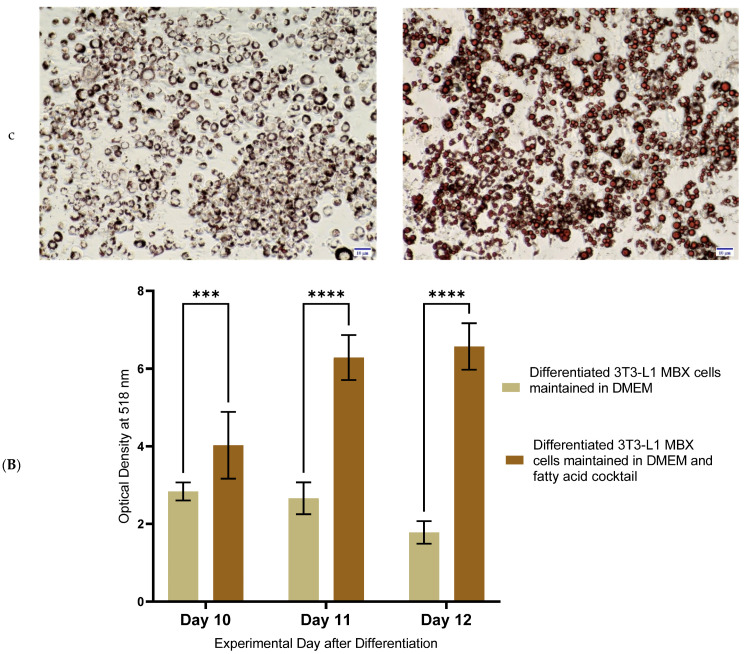
Analysis of accumulated lipids in differentiated 3T3-L1 MBX cells with and without the combination treatment of the 3 fatty acids cocktail in DMEM after the initiation of differentiation from Day 6. (**A**) Representative 20X images of non-oil red O stained (a) and oil-red O stained (b,c), Differentiated 3T3-L1 MBX cells cultured in DMEM in the presence and absence of fatty acids cocktail on Day 12 post-initiation of differentiation. (**B**) Quantitation of accumulated lipids in differentiated 3T3-L1 MBX on Days 10, 11, and 12. Each data point represents the average measure of optical density of oil-red O stain using a plate reader in 3 replicates for 3 independent experiments. The statistical significance was derived using Two-way ANOVA, and Tukey’s post hoc test was employed for multiple comparisons of group means in between. A *p*-value < 0.05 was considered statistically significant. Three asterisk (***) indicates *p* ≤ 0.001, and four asterisk (****) indicates *p* ≤ 0.0001.

To better reflect the effects of increased serum fatty acid concentrations and their exposure to peripheral tissues in obesity, differentiated 3T3-L1 MBX white adipocyte cells were maintained in the presence or absence of a fatty acids cocktail (oleic acid, linoleic acid, and palmitic acid). Changes in their lipid accumulation profiles were then determined. Results are summarized in Figure 4. 3T3-L1 MBX cells were grown to ~80% confluence and then were differentiated with methyl isobutyl xanthine (0.5 mM); dexamethasone (1 µM); insulin (10 µg/mL); rosiglitazone (2 µM) for 3 days. On Day 3, differentiation components containing DMEM media were discarded and replaced with maintenance media containing 10 µg/mL concentration of insulin in DMEM for 3 days. From Day 6 onwards, 3T3-L1 MBX cells were maintained in DMEM in the presence or absence of a fatty acids cocktail in the dose range determined by MTT results. On Days 10, 11 and 12, their lipid accumulation profiles were analyzed and compared using oil-red O stain, which stains lipids (Figure 4). Images of the differentiated 3T3-L1 MBX cells stained with oil-red O demonstrated that the mean proportion of differentiated 3T3-L1 MBX cells with accumulated lipids was greater when they were maintained in DMEM with the fatty acids cocktail in comparison to differentiated 3T3-L1 MBX cells cultured in DMEM without the cocktail. Quantitative analysis of the accumulated oil-red O stain by plate reader indicated that differentiated 3T3-L1 MBX cultured in DMEM with the fatty acids cocktail had a 2-fold increase in lipid accumulation on Day 10 and more than 4-fold increase in lipid accumulation on Days 11 and 12 in comparison to differentiated 3T3-L1 MBX cells cultured in DMEM without the fatty acids cocktail.

### 3.5. Differentiation in the Presence of the Fatty Acids Cocktail Altered the Secretome Profile of 3T3-L1 MBX Cells

**Figure 5:**
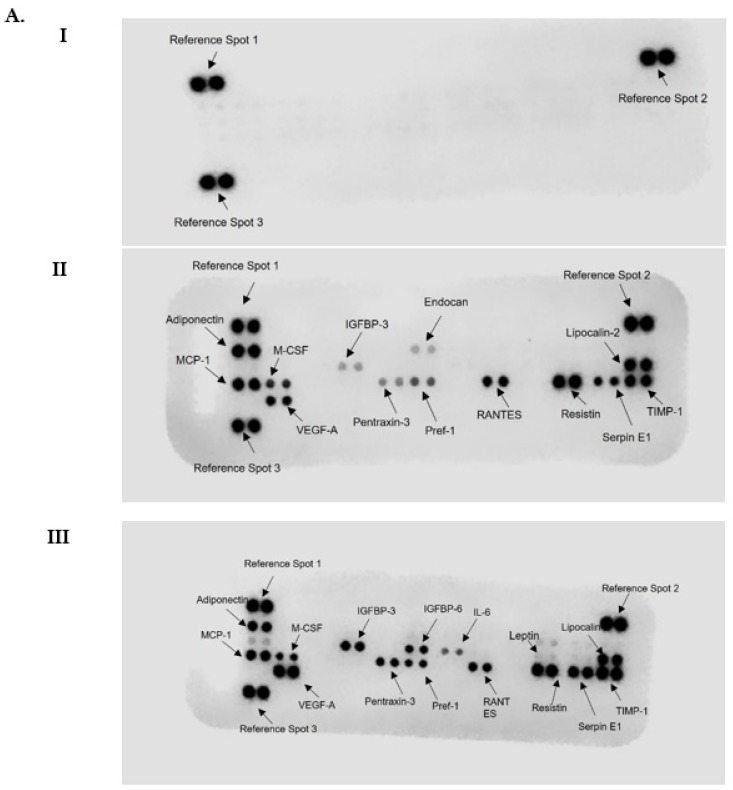

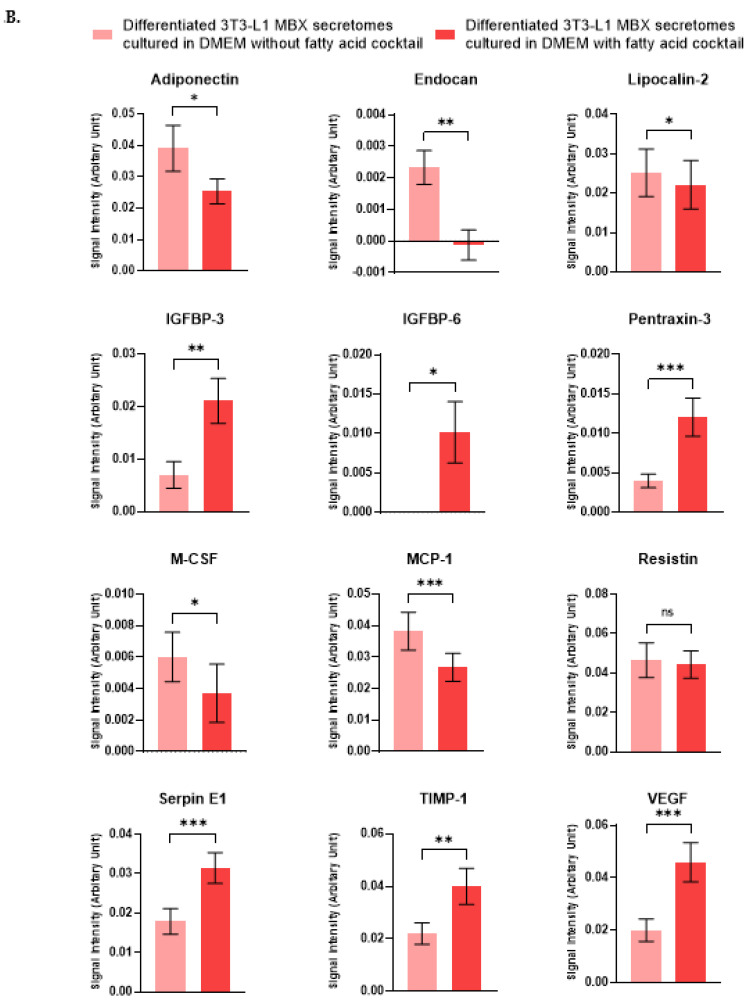

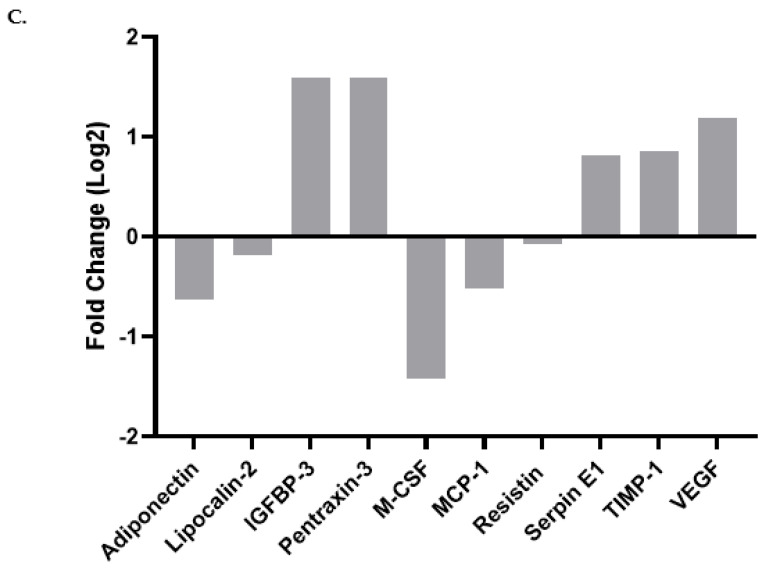
Adipocyte secretions from differentiated 3T3-L1 MBX cells on Day 11 with and without the combination treatment of 3 kinds of fatty acids in DMEM after the initiation of differentiation from Day 6. (**A**) The dot blots indicate different concentrations of adipokines detected on nitrocellulose membrane after treatment with supernatants of (I) DMEM media without FBS, which has been used for conditioning cells (II) 3T3-L1 MBX cells from Day 11 treated with FBS-free DMEM in the absence of fatty acids cocktail (III) 3T3-L1 MBX cells from Day 11 treated with FBS-free DMEM in the presence of fatty acids cocktail. (**B**) Bar graphs of adipokines with the observable changes in adipokine secretions from differentiated 3T3-L1 MBX cells cultured in the presence or absence of fatty acids cocktail. Each data point represents the mean value of the secretome in 2 technical replicates for 3 independent experiments. (**C**) Fold change expression of secreted adipokines from differentiated 3T3-L1 MBX cells on Day 11 treated with fatty acids cocktail in respect to differentiated 3T3-L1 MBX cells on Day 11 treated without fatty acids. The statistical significance between the treatment group and the control was determined using a paired *t*-test. A *p*-value < 0.05 was considered statistically significant. One asterisk (*) indicates *p* ≤ 0.05, two asterisk (**) indicates *p* ≤ 0.01, and three asterisk (***) indicates *p* ≤ 0.001. *p*-value > 0.05 was considered statistically nonsignificant (ns).

Differentiated 3T3-L1 MBX murine cells are a recently developed in vitro model for studying white adipocytes. Here we have shown that when differentiated in the presence of a fatty acids cocktail, 3T3-L1 MBX cells chronologically increased lipid accumulation on Days 10, 11, and 12 post-differentiation. Based on previously reported results, we expected that changes in white adipocyte secretions would occur due to increased fat accumulation [5]. To that end, the adipocyte secretome of 3T3-L1 MBX cells from Day 11 was analyzed post-differentiation in the presence or absence of fatty acids cocktail using a proteome profiler mouse adipokine array kit. The results are shown in Figure 5 and confirm our hypothesis that significant changes exist in the 3T3-L1 MBX adipocyte secretome based on fatty acids cocktail exposure. Table 2. summarizes the calculated mean differences ± SEM (standard error of the mean) and significances of the changes (using paired *t*-test, *p* < 0.05) in selected secreted adipokines from differentiated 3T3-L1 MBX cells cultured in the presence or absence of the fatty acids cocktail. The data demonstrated that differentiated 3T3-L1 MBX, when cultured in the presence of fatty acids cocktail, there was found to have increased secretion of adipokines such as IGFBP-3, IGFBP-6, Serpin-E1, VEGF, TIMP-1, Pentraxin-3 and decreased secretion of adipokines, such as MCP-1, M-CSF, Adiponectin, Lipocalin-2 in comparison to differentiated 3T3-L1 MBX, when cultured in absence of fatty acids cocktail. Figure 5A shows the visualized changes of each adipokine on the dot blot membrane; using a bar graph (Figure 5B); and the calculated fold change (Figure 5C) value.

### 3.6. Expression of Key Marker of Adipogenesis in 3T3-L1 MBX Cells from Its Journey from Preadipocyte to Adipocyte Lineage w/o the Treatment of Fatty Acid

**Figure 6:**
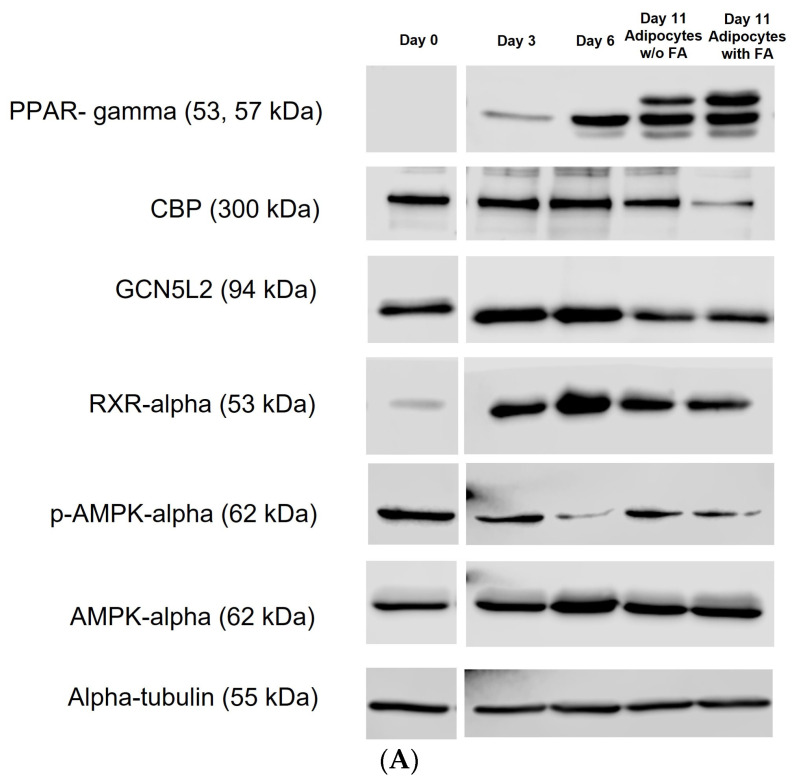

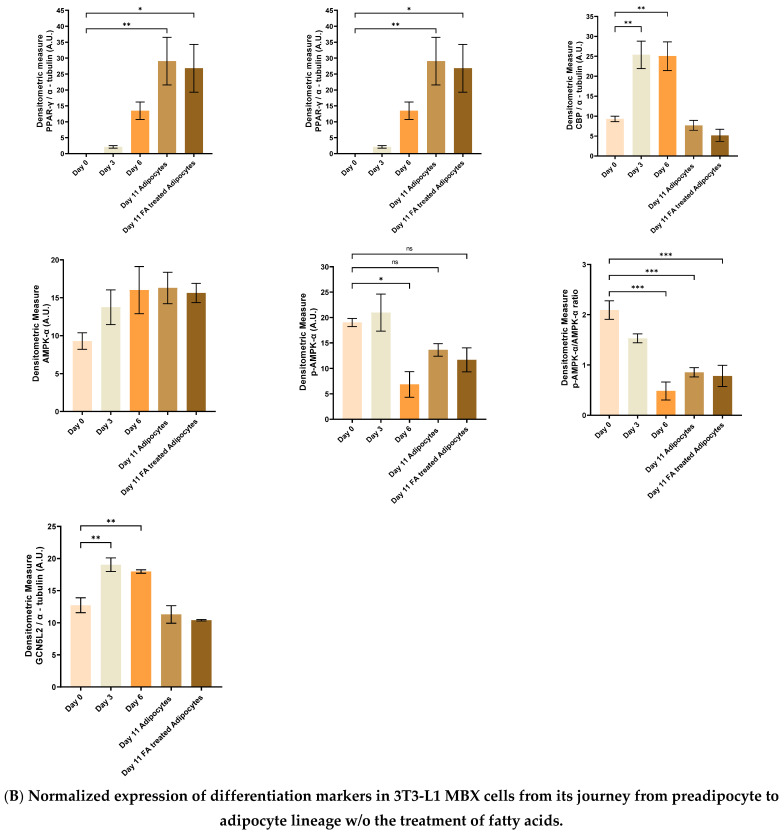
The expression of adipogenesis-related proteins in 3T3-L1 MBX cells from its journey from preadipocyte to adipocyte lineage w/o the treatment of fatty acids cocktail. (**A**) Immunoblotting image and (**B**) protein expression level of adipogenesis-related protein in 3T3-L1 MBX cells on Day 0, Day 3, Day 6, and Day 11 (with and without the fatty acids cocktail treatment). Each data point represents the average measure of densitometric quantification of protein expression for 3 independent biological replicates for each Day of protein extract. The statistical significance was derived using One-way ANOVA, and Dunnett’s multiple comparison test was employed for comparing the expression of regulatory proteins in differentiated days (Day 3, 6, and 11) to Day 0 when the 3T3-L1 MBX cells were in non-differentiated condition. A *p*-value < 0.05 was considered statistically significant. One asterisk (*) indicates *p* ≤ 0.05, two asterisk (**) indicates *p* ≤ 0.01, and three asterisk (***) indicates *p* ≤ 0.001. *p*-value greater than 0.05 was considered statistically non-significant (ns). Here: FA: fatty acid; PPAR-γ: Peroxisome Proliferator- Activated Receptor gamma; CBP: CREB binding protein; GCN5L2: General Control of Amino Acid Synthesis Yeast Homolog Like 2; AMPK-α: AMP-Activated Protein Kinase, RXR-α: cis-Retinoic Acid Receptor.

The expression of a wide range of regulatory proteins at the molecular level controls the ability of adipocytes to develop from preadipocyte lineage. The expression of those proteins has been extensively studied for the 3T3-L1 cell line to obtain an overview of proteins controlling adipocyte biology at the molecular level. The present study has explored the expression of some of the regulatory proteins controlling the development of adipocytes to their maintenance in 3T3-L1 MBX cells. The study has also examined the changes in the expression of those proteins in adipocyte lineages in the presence and absence of the fatty acids cocktail treatment. 3T3-L1 MBX cells were grown to ~80% confluence and then were differentiated with methyl isobutyl xanthine (0.5 mM); dexamethasone (1 µM); insulin (10 µg/mL); rosiglitazone (2 µM) for 3 days (see above). On Day 3, differentiation components containing DMEM media were discarded and replaced with maintenance media containing 10 µg/mL concentration of insulin in DMEM for three days. From Day 6 onwards, 3T3-L1 MBX cells were maintained in DMEM in the presence or absence of a fatty acids cocktail in the dose range determined by MTT results. Whole-cell protein extract from 3T3-L1 MBX cells has been collected on Day 0, Day 3, Day 6, and Day 11 (treated w/o fatty acids cocktail). Proteins were then quantified and explored for the expression of adipogenesis or fat cell differentiation marker proteins PPAR-γ, CBP, GCN5L2, p-AMPK-α, and RXR-α (Figure 6).

## 4. Discussion

Access to an accurate and reliable in vitro model to investigate the effects of adipocyte secretions and the influence of obese cells on neighboring cells and tissues is needed to better understand the impact of obesity and obesity-driven inflammation on both cancer cells and normal cells. Currently, one of the most used cell lines is 3T3-L1. Here, we analyzed the efficiency and characteristics of its clonal derivative, the 3T3-L1 MBX cell line. We observed and reported increased differentiation yields as well as lipid accumulation and adipokine status when their differentiation was maintained in the presence of fatty acids cocktail (not previously reported). Cells were exposed to an optimized differentiation cocktail of methyl isobutyl xanthine, dexamethasone (1 µM), insulin (10 µg/mL), and rosiglitazone for 3 days. During this time, cells transitioned from the fibroblast phenotype to an adipocyte phenotype and acquired the capability of accumulating lipids. The capacity of accumulating lipids in droplets comes from the insulin-mediated uptake of glucose and fatty acid from the exogenous media, followed by activation of de novo lipogenesis [39,40]. With the exogenous treatment of fatty acids, 3T3-L1 MBX cells were observed to become oversaturated with lipids due to increased availability of fatty acids, followed by their uptake and esterification to fat droplets in comparison to control. The accumulation of lipids has been attributed to both the activation of de novo lipogenesis events and the continuous uptake of exogenous fatty acids from the culture medium post-differentiation.

In this study, the concentration of the differentiation components, dexamethasone and insulin, were analyzed at supraphysiological levels to differentiate 3T3-L1 MBX fibroblast cells to mature adipocytes. Although the physiological doses of each drug and the saturation point of their glucocorticoid and insulin receptors, respectively, are in the nanomolar range, the study has increased their dose range into the micromolar range. These pharmacological concentrations were used to ensure terminal adipogenesis of the maximum number of 3T3-L1 MBX fibroblast cells (preadipocytes). As more cells achieve terminal adipogenesis, they are capable of accumulating lipids. Moreover, the study hypothesized that the excessive accumulation of lipids due to exogenous fatty acid availability would be followed by changes in the adipocytokine secretions. These changes were predicted to more closely mimic in vivo obesity. Hence, this pharmacological intervention for dexamethasone and insulin can be viewed as a “synthetic model” that recreates physiological responses under artificial conditions.

Analysis of the secretion profiles of 3T3-L1 MBX adipocyte cells, when they are exposed to the fatty acids cocktail, revealed that there were changes in the release of key adipokines in comparison to the control differentiated cells with no fatty acids added. One of these key adipokines is adiponectin. Not only is it the most abundant adipokine secreted from the adipose tissue [41], but it also plays a protective role in minimizing the effects of inflammation, maintaining whole-body energy homeostasis by expediting lipid and carbohydrate metabolism, increasing insulin sensitivity for the glucose transport system, as well as inhibiting hepatic gluconeogenesis and lipogenesis [42,43,44,45,46]. Our results showed that when 3T3-L1 MBX cells were treated with the fatty acids cocktail, expression of adiponectin was significantly decreased. This outcome is in line with epidemiological and experimental evidence in obesity studies that showed decreased expression of adiponectin correlates with the increase in fat mass [47,48,49].

Another pair of important adipokines that significantly increased in the presence of fatty acids were insulin-like growth factor binding protein 3 and 6 (IGFBP-3 and IGFBP-6). These binding proteins are involved in insulin like growth factor (IGF) binding and inducing their biological effect [50]. However, the action of these proteins can also be independent of the IGF–IGFR-induced signaling pathway [50]. Data from several studies have concluded IGFBP-3 is elevated in obesity and has a role in insulin resistance, glucose intolerance, and less glucose clearance [51,52,53]. The expression of lipocalin-2 was reduced in 3T3-L1 MBX cells cultured with the fatty acids cocktail, in contrast to the observations reported from previous studies where it has been demonstrated to have a link between the increased expression of lipocalin 2 with insulin resistance, obesity, and hyperglycemic conditions [54,55,56]. Lipocalin 2 is a circulatory adipocytokine responsible for transporting hydrophobic molecules like steroid hormones, fatty acids, etc., to their target site of action [57]. Its expression is highly regulated by nutritional stress and inflammation [56]. The reduced expression of Lipocalin 2 from the 3T3-L1 clone treated with the combination fatty acids cocktail in comparison to the control can be attributed to the clone itself; the day is chosen after differentiation to evaluate the adipokine secretions or due to the high non-toxic concentration of fatty acid treatment in combination. Adipocytes also secrete chemokines as an endocrine factor. MCP-1 is a chemokine responsible for attracting blood monocytes to the site of inflammation [58]. M-CSF is a growth factor cytokine, mediating local survival, proliferative, and differentiating signaling to the mononuclear phagocytic cells at the site of inflammation [59]. The expression levels of MCP-1 and M-CSF were also significantly reduced in differentiated 3T3-L1 MBX cells in the presence of the fatty acids cocktail.

The remaining adipokines pentraxin-3 [60,61], Serpin E1 [62,63], TIMP-1 [64,65], and VEGF [66,67] all demonstrated significantly higher expression levels in 3T3-L1 MBX cells treated with the fatty acids cocktail compared to controls. This increase in expression is similar to previous studies reporting their increased expression under obese conditions, along with their association with obesity-driven comorbid diseases [67,68,69,70,71,72,73,74,75]. Pentraxin-3, an inflammatory biomarker, is secreted under inflammatory conditions, which were further found to promote cell stemness [76] and metastasis [75,77]. Serpin E1 is a plasminogen activator inhibitor encoded by the SERPIN E1 gene, which primarily plays a role in the stabilization of fibrinogen clot formation and maintenance of wound healing [78]. However, studies have found Serpin E1 signaling to have a role in malignant progression and resistance [79,80,81,82], hepatic steatosis [69], and aging [83]. TIMP-1 belongs to the family of tissue inhibitors of metalloproteinases and physiologically plays a role in the inhibition of matrix metalloproteinase that, in turn, causes degradation of the extracellular matrix (ECM) and tissue remodeling [84]. With increasing TIMP-1 secretion under fatty acid-treated conditions, adipocytes can expand and accumulate more fat by TIMP1-mediated remodeling of the extracellular matrix [85]. TIMP-1 can also act as a signaling molecule on the cell surface receptor CD63, activating downstream signaling pathways and paving the way for cancer pathogenesis [86,87,88]. Vascular endothelial growth factor (VEGF) primarily functions in neovascularization and vascular permeability through VEGFR-mediated signaling in endothelial cells [89]. This angiogenic effect promotes the metastatic progression of cancer [90]. Apart from the adipokines listed above, conditioned media collected from differentiated 3T3-L1 MBX cells treated with fatty acids cocktail on Day 11 was found to have inflammatory cytokine IL-6 present on the membrane in comparison to the conditioned media collected from differentiated 3T3-L1 MBX cells without the fatty acids cocktail treatment. This outcome is also in line with the evidence in obesity studies that showed increased expression of IL-6 correlates with the increase in fat mass [91,92]. IL-6 is a proinflammatory cytokine that conduces local and systemic inflammation [93], associated with clinical events of insulin resistance [94,95], cancer metastasis [96,97], and other inflammatory metabolic disorders.

Analysis of the expression of the regulatory proteins controlling the adipogenesis events of 3T3-L1 MBX cells in response to the treatment using differentiation components and later fatty acids cocktail gives us a molecular overview of the successful differentiation and maintenance of adipogenesis characteristics in 3T3-L1 MBX adipocytes. PPAR-γ, a master regulator of terminal adipogenesis, imparts a vital role in adipocyte biology by transcriptionally regulating the physiological process from their development to the maintenance of metabolism [98,99]. It is a member of the nuclear receptor superfamily of ligand-activated transcription factors [100]. It exerts its biological effect through forming a heterodimer with cis-retinoic acid receptor alpha isotype (RXR-α) with subsequent recruitment of coactivators upon ligand binding, following assemble at PPAR response element (PPRE) in the promoter region resulting in transactivation of target genes regulating differentiation to adipocytes, maintenance of adipocytes through lipid droplet formation, glucose uptake (GLUT4), improving insulin sensitivity, fatty acid transport (FABP4, CD36) and storage, de novo lipogenesis [99,100,101,102,103,104]. In this study, we have found fibroblast form of 3T3-L1 MBX has no expression for PPAR-γ protein on Day 0. With the hormonal stimulation with differentiation cocktail 3T3-L1 MBX cells start expressing PPAR-γ protein from Day 3 with a gradual increase in the expression level from Day 6 to Day 11, both in the presence and absence of fatty acids cocktail treatment. It can be interpreted as the PPAR-γ protein playing a role in the terminal differentiation of 3T3-L1 MBX adipocytes by inducing gene expression responsible for maintaining functional adipocyte and metabolic equilibrium. With the fatty acid treatment in this study, the PPAR-γ expression in differentiated 3T3-L1 MBX cells does not change much with respect to differentiated 3T3-L1 MBX cells that have not been treated with fatty acid. With this, we can conclude that fatty acid treatment on differentiated 3T3-L1 MBX cells does not hamper the functionality of differentiated 3T3-L1 MBX cells. Instead, it makes the fat cell accessible to the monomer of fatty acid and, with the PPAR-γ mediated responsible gene expression, accumulates the fatty acid monomer in lipid droplets as triglycerides, followed by more lipid accumulation. PPAR-γ imparts its effect by forming a heterodimer with the RXR-α receptor. Our study found increased expression of RXR-α receptor in 3T3-L1 MBX cells after the hormonal stimulation. This statement also remains consistent with the observation found for PPAR-γ in this study, maintenance of intact functionality of differentiated 3T3-L1 MBX adipocytes through mediating the expression of adipocyte-specific genes.

cAMP-regulated transcription factor, cAMP-responsive element binding protein (CREB), has been implicated as an initiator of adipocyte differentiation program [21]. CREB binding protein (CBP) coactivates many transcriptional activators recruited to the target gene promoter [105]. Following the stimulation of preadipocytes with a differentiation cocktail, the rise of intracellular cyclic adenosine monophosphate (cAMP) promotes the activation of protein kinase A (PKA) [21]. The activated catalytic subunit then translocates to the nucleus and phosphorylates CREB protein at serine 133 [21]. PKA-mediated activation of CREB protein results in its binding to a nuclear protein CBP which acts as a coactivator for CREB [21]. The regulatory unit of the proximal promotor of CCAAT Enhancer-Binding protein β (C/EBP-β) has a core binding site for CREB, and this is how CBP protein plays a role in the initial phases of adipogenesis through regulating the expression of C/EBP-β transcriptional factor [21]. Our study observed CBP protein expression in preadipocytes followed by a gradual increase in its expression in differentiated adipocytes until Day 6 post-differentiation. Later, the expression of CBP decreases in mature adipocytes on Day 11, irrespective of fatty acid treatment. From this observation, it can be concluded that constant expression of CBP in the initial phase of adipocyte differentiation speaks for the successful initiation of adipogenesis in 3T3-L1 MBX cells with the changed concentration of differentiation components in the differentiation cocktail. We have also explored the expression of another protein GCN5L2 in preadipocyte and differentiated adipocytes. GCN5L2 is a histone acetyltransferase enzyme that has been found to remain upstream and regulate the expression of PPAR-γ during adipogenesis [106]. In this study, GCN5L2 protein expression was found to be significantly increased with the induction of differentiation in 3T3-L1 MBX cells on Day 3 and Day 6, followed by a decrease in their expression in differentiated adipocytes on Day 11 w/o the fatty acids treatment. It can be interpreted as GCN5L2 expression, by remaining upstream, has successfully handled the gradually increased expression of PPAR-γ from the initial days of adipogenesis.

AMP-activated protein kinase (AMPK-α) is a cellular energy-sensing protein kinase that’s regulates energy metabolism by activating catabolic pathways and inhibiting anabolic pathways [107,108]. Studies have also found that AMPK activation inhibits the differentiation event in preadipocytes by blocking the activity of transcriptional factor PPAR-β, followed by stopping the terminal adipogenic events in adipocytes [108,109,110,111]. Studies have also found that AMPK activation inhibits the differentiation event in preadipocytes by blocking the activity of transcriptional factor PPAR-β, followed by stopping the terminal adipogenic events in adipocytes [108,109,110,111]. In the study, the preadipocyte form of 3T3-L1MBX has been found to have enhanced activation of AMPK-α through phosphorylation. In the presence of hormonal stimulation with a differentiation cocktail, the differentiated state of 3T3-L1 MBX has been found to have a gradual decrease in the phosphorylation of AMPK-α from Day 3 to Day 6, followed by a decrease in the maintenance of phosphorylation status of AMPK-α in differentiated adipocytes on Day 11 irrespective of the fatty acids cocktail treatment. This observation can be interpreted as with the hormonal stimulation for the differentiation of 3T3-L1 MBX cells, the decreased activation of AMPK stimulates de novo lipogenesis, energy storage through triglyceride deposition, adipogenic events in adipocytes, speaking off maintenance of adipogenesis in 3T3-L1 MBX cells with the changed concentration of differentiation components and w/o the fatty acids cocktail treatment.

Overall, the study exploring regulatory protein expression related to adipogenesis gives us an overview of the successful adipogenic conversion of 3T3-L1 MBX preadipocytes to adipocyte lineage. Also, it provides us with an understanding of how adipocytes accumulate lipids in the presence and absence of fatty cocktail treatment. Based on the expression of the protein above and their association with the activation of the related signaling pathway, it can be interpreted that when the cells are not treated with a fatty acids cocktail, the accumulation of lipids in differentiated 3T3-L1 MBX cells is mediated by uptake of glucose from the media and then the conversion of glucose to fatty acids via de novo lipogenesis. These fatty acids gets esterified and stored as triglycerides. When the cells are treated with a fatty acids cocktail, with de novo lipogenesis-mediated conversion of glucose to fatty acids, excess fatty acids from the media are up taken, transported, and esterified to triglycerides in lipid droplets causing excessive lipid accumulation.

Obesity is endocrinologically responsible for the physiological consequences of less insulin sensitivity followed by hyperinsulinemia, glucose intolerance, proinflammation, hepatic steatosis, dyslipidemia, tumorigenicity, and metastasis [112]. The development of obesity needs continuous differentiation of non-adipogenic fibroblasts to adipocytes. Data obtained for the secreted adipokines in 3T3-L1 MBX cells cultured with the fatty acids cocktail have demonstrated it to be more reflective of the metabolic complications seen in obesity versus differentiated 3T3-L1 MBX cells not exposed to available fatty acids. While our results demonstrate successful and impactful changes in the 3T3-L1 MBX cells under optimized differentiation and obesity-associated conditions, limitations to the study need to be mentioned. One of the limitations of characterization was selecting only the 11th day after the initiation of differentiation of 3T3-L1 MBX cells for evaluating their adipokine profiles. This may have underestimated the fat accumulation and secretome profile changes of differentiated 3T3-L1 MBX cells if they were evaluated later than Day 11 post differentiation (ex: Day 14, Day 16, Day 18). Moreover, a slight appearance of leptin expression or other proinflammatory cytokines (IL-6) was observed in the secretomes of differentiated 3T3-L1 MBX cells treated with a fatty acids cocktail. However, the appearance of dots is not strong enough to be measured. I assume using the ELISA kit for Leptin, IL-6 expression exploration would have given more concrete information on the level of expression of these adipokines. Examples of future studies include measuring the triglycerides, reactive oxygen species, fatty acid binding protein-4 (FABP4), proteins related to lipogenesis and lipolysis (Cav-1, SREBP-1c, PLIN1, fatty acid synthase (FASN), and hormone sensitive lipase (HSL), among other to amass a more comprehensive overview of fatty acid-induced changes in these obese adipocytes. All the above-mentioned studies can also be performed in future studies with human white adipocytes to establish an in vitro human adipocyte fat cell model that more closely mimics obesity.

## 5. Conclusions

In this study, the differentiation signaling compounds’ concentrations were optimized to produce the greatest percentage of differentiation in the fibroblast cell line, 3T3-L1 MBX. That final mixture was 0.5 mM methyl isobutyl xanthine; 1.0 µM dexamethasone; 10 µg/mL insulin; 2 µM rosiglitazone. In addition, the time was optimized to 3 days for differentiation. The yield under these conditions was >90%. Furthermore, to generate obese adipocytes, fatty acids supplementation was added as a cocktail of 0.75 mM oleic acid, 0.85 mM linoleic acid, and 0.1 mM palmitic acid. Under these conditions, the 3T3-L1 MBX cells differentiated into adipocytes and then accumulated fats significantly greater (4X) than cells cultured without the fatty acids cocktail. Their lipid accumulation and changes in the secretion of adipokines were determined. Moreover, the exploration of regulatory protein expression in differentiated 3T3-L1 MBX cells has also confirmed successful differentiation and maintenance of adipogenesis events in 3T3-L1 MBX cells with the changed concentration of differentiation components and irrespective of fatty acids cocktail treatment. In conclusion, our study of 3T3-L1 MBX cells cultured with the fatty acids cocktail demonstrates the utility of this system as an in vitro obese fat cell model investigating the downstream molecular effects of obesity-associated metabolic disorder and more closely resembling obese fat cells and their lipid deposits in vivo.

## Figures and Tables

**Table 1:** Details of Differentiation Compounds Used in Adipocyte Differentiation.

Differentiation Compounds	Bio-Signaling of Differentiation Compounds in Adipogenesis	Conc. Used for 3T3-L1 MBX Differentiation
Methyl Isobutyl Xanthine	Inhibits phosphodiesterase and increases the expression of cAMP; cAMP then phosphorylates and activates CREB, which further induces endogenous expression of C/EBPβ to promote adipogenesis [24]	0.5 mM
Dexamethasone	Stimulates the expression of the adipogenic markers PPARγ and C/EBPα; attenuates Pref-1 expression [24]	1 µM
Insulin	(a) Acts as a growth factor through IGF-1-mediated pathway and regulates fibroblast differentiation by increasing the expression of fat-specific transcription factors, including SREBP-1c and PPARγ through PI3K and Akt signaling pathway [24,25] (b) Stimulates the de novo fatty acid synthesis in adipocytes [25] (c) Facilitates the uptake of glucose and fatty acid in adipocytes [25]	10 µg/mL
Rosiglitazone	PPAR-γ agonist and therefore helps in promoting the PPAR-γ mediated expression of genes related to terminal adipogenesis [26]	2 µM

Abbreviations: SREBP-1c: Sterol regulatory element binding protein 1, PPARγ: Peroxisome proliferator-activated receptor gamma, C/EBP: CCAAT-enhancer binding protein alpha, CREB: cAMP-responsive element binding protein, Pref-1: Preadipocyte factor 1.

**Table 2:** Calculated measure of difference in adipokine secretions from differentiated 3T3-L1 MBX cells cultured in the presence or absence of the fatty acids cocktail.

Adipokines	Differentiated 3T3-L1 MBX Secretome Cultured without the Fatty Acids Cocktail. (Mean ± SEM)	Differentiated 3T3-L1 MBX Secretome Cultured with the Fatty Acids Cocktail. (Mean ± SEM)	*p*-Value	Significance
Adiponectin	0.039130374 ± 0.002316738	0.025347322 ± 0.000861227	0.0264	Yes
Endocan	0.002328695 ± 0.000169974	−0.000200098 ± 0.000847013	0.0040	Yes
Lipocalin-2	0.025215261 ± 0.001906274	0.022184057 ± 0.000788183	0.0413	Yes
IGFBP-3	0.006996293 ± 0.000803687	0.021083799 ± 0.000814481	0.0083	Yes
IGFBP-6	0	0.010152521 ± 0.000807328	0.0287	Yes
Pentraxin-3	0.003977768 ± 0.000273779	0.012053091 ± 0.000667569	0.0007	Yes
M-CSF	0.006015142 ± 0.000496422	0.002255953 ± 0.000721472	0.0423	Yes
MCP-1	0.038259467 ± 0.001904069	0.026713838 ± 0.000766827	0.0001	Yes
Resistin	0.046562 ± 0.002796748	0.044354171 ± 0.000445862	0.6767	No
SERPIN E1	0.017874348 ± 0.001031738	0.031444413 ± 0.000445274	0.0003	Yes
TIMP-1	0.021979934 ± 0.001289831	0.040024761 ± 0.000436769	0.0027	Yes
VEGF	0.020019044 ± 0.001350236	0.045899346 ± 6.71564 × 10^−5^	0.0004	Yes

## Data Availability

Data is contained within the article.
